# Transcriptional Profiles of *SmWRKY* Family Genes and Their Putative Roles in the Biosynthesis of Tanshinone and Phenolic Acids in *Salvia miltiorrhiza*

**DOI:** 10.3390/ijms19061593

**Published:** 2018-05-29

**Authors:** Haizheng Yu, Wanli Guo, Dongfeng Yang, Zhuoni Hou, Zongsuo Liang

**Affiliations:** 1 Institute of Soil and Water Conservation, Chinese Academy of Sciences (CAS) & Ministry of Water Resources (MWR), Yangling 712100, China; yuhz@nwafu.edu.cn; 2 Zhejiang Province Key Laboratory of Plant Secondary Metabolism and Regulation, Zhejiang Sci-Tech University, Hangzhou 310018, China; gwl1016@aliyun.com (W.G.); yangdongfeng@zstu.edu.cn (D.Y.); houzhuoni@zstu.edu.cn (Z.H.); 3 College of Life Science, Zhejiang Sci-Tech University, Hangzhou 310018, China; 4 University of Chinese Academy of Sciences, Beijing 100049, China

**Keywords:** *Salvia miltiorrhiza*, *SmWRKY*, secondary metabolites, tanshinones, phenolic acid

## Abstract

*Salvia miltiorrhiza* Bunge is a Chinese traditional herb for treating cardiovascular and cerebrovascular diseases, and tanshinones and phenolic acids are the dominated medicinal and secondary metabolism constituents of this plant. WRKY transcription factors (TFs) can function as regulators of secondary metabolites biosynthesis in many plants. However, studies on the WRKY that regulate tanshinones and phenolics biosynthesis are limited. In this study, 69 *SmWRKYs* were identified in the transcriptome database of *S. miltiorrhiza*, and phylogenetic analysis indicated that some *SmWRKYs* had closer genetic relationships with other plant *WRKYs*, which were involved in secondary metabolism. Hairy roots of *S. miltiorrhiza* were treated by methyl jasmonate (MeJA) to detect the dynamic change trend of *SmWRKY*, biosynthetic genes, and medicinal ingredients accumulation. Base on those date, a correlation analysis using Pearson’s correlation coefficient was performed to construct gene-to-metabolite network and identify 9 SmWRKYs (SmWRKY1, 7, 19, 29, 45, 52, 56, 58, and 68), which were most likely to be involved in tanshinones and phenolic acids biosynthesis. Taken together, this study has provided a significant resource that could be used for further research on SmWRKY in *S. miltiorrhiza* and especially could be used as a cue for further investigating SmWRKY functions in secondary metabolite accumulation.

## 1. Introduction

*Salvia miltiorrhiza* Bunge is a well-known Chinese herb with significant medicinal and economic value. The root of *Salvia miltiorrhiza*, which is called “danshen” or “tanshen” in Chinese, has been used primarily in traditional Chinese medicine (TCM) for clinical treatment of coronary heart, menstrual disorders, cardiovascular diseases, acute ischemic stroke diseases, and inflammation [1]. The major bioactive constituents of *S. miltiorrhiza* are classified into two categories: lipophilic tanshinones and hydrophilic phenolic acids [2]. More than 40 tanshinones (tanshinone I, tanshinone IIA, cryptotanshinone, dihydrotanshinone I, and so on) and 20 hydrophilic phenolic acids (salvianolic acid B, rosmarinic acid, dihydroxyphenyllactic acid, and lithospermic acid) have been isolated and identified from *S. miltiorrhiza* [3]. Phenolic acids in *S. miltiorrhiza* are biosynthesized through the phenylpropanoid and tyrosine-derived pathways. It has been suggested that salvianolic acid B (SAB) is derived from the rosmarinic acid (RA) pathway. The biosynthetic pathway of rosmarinic acid (RA) is well characterized in plants [4,5]. Tanshinones in *S. miltiorrhiza* are produced through the mevalonate (MVA) and methylerythritol phosphate (MEP) pathways, and the biosynthetic pathway of ferruginol is well characterized. Most of the key biosynthetic enzyme genes of those pathways have been cloned [6,7,8,9,10,11]. However, the regulatory mechanisms of tanshinones and phenolic acids biosynthesis are largely unresolved.

Tanshinones and phenolic aicds were major secondary metabolites in *S. miltiorrhiza.* Many secondary metabolites play important roles in the biology of pants; for example, anthocyanins are pigments in fruits and flowers, generating colorful form and thereby conferring the quality of fruits [12]. In tomato plants, quercetin derivatives were dramatically increased in response to low temperatures when under conditions of nitrogen starvation, and especially at higher light intensities [13]. In addition, a cold-acclimation acquisition olive-tree, named Canino, produces more unsaturation and cutinisation than cold-sensitive genotypes [14]. For people, plant secondary metabolites, especially phenolic acid, are important health-promoting pigments because of their potential antiradical (scavenging) activities [15]. Transcription factors play a central role in the progress of plant secondary metabolite biosynthesis [16]. For example, *SmMYB39* repressed phenolic acids biosynthesis when overexpressed in *S. miltiorrhiza* [17], whereas SmPAP1, the other member of MYB TFs, is involved in enhancing phenolic acid production by interacting with bHLH TFs SmMYC2 and activating genes encoding key enzymes in *S. miltiorrhiza* Bge. f. alba [18]. Zhou et al. found JAZ-interacting TFs SmMYC2a and SmMYC2b positively regulate the biosynthesis of tanshinones and salvianolic acid B [19]. In addition to this TFs-regulated phenolic acid production, the basic leucine zipper (bZIP) transcription factor *OsTGAP1* that was overexpressed in rice can hyperaccumulate momilactones and phytocassanes, which function as antimicrobial secondary metabolites in response to pathogen attacks [20]. *OsbZIP79* interacted with *OsTGAP1* and was involved in the regulation of phytoalexins production [21]. TPS10 is a terpene synthase; the AP2/ERF transcription factor, EREB58, positively regulates sesquiterpene production by directly promoting *TPS10* expression [22].

The WRKY is the 7th largest TFs family in flowering plants following the bHLH, MYB, ERF, NAC, bZIP, and C2H2 families [23]. The role of WRKY responds to abiotic and biotic stresses, such as wounding, drought, heat, and pathogens; these topics that have been of extensive concern recently [24,25,26,27,28,29]. In addition to the response to biotic and abiotic stresses, accumulating evidence indicates that WRKYs also play an essential role in secondary metabolism production by regulating the related biosynthetic genes. For phenolic acid biosynthesis, *PtrWRKY19* from *Populus trichocarpa* led to the repression of the lignin biosynthesis-related genes when they were overexpressed in transgenic plants [30]. Moreover, *Vitis vinifera* L. VvWRKY2, which was transferred into tobacco, exhibited altered, related gene expression in the lignin pathway and cell wall formation by activating the promoter of the *VvC4H* gene, which is one of the lignin biosynthetic pathway genes and was confirmed by transient transcriptional activation assays in tobacco protoplasts [31]. Similar to these WRKYs that are involved in the phenolic acid biosynthetic pathway, some WRKY-regulating terpenoid pathways also have been reported recent years. For example, over-expression of *OsWRKY56* can induce diterpenoid phytoalexins (momilactones) production by up-regulating *OsCPS4* and *OsCYP99A2*, which encoded biosynthetic enzymes of the rice diterpenoid pathway [32]. *PqWRKY1*, one of the *WRKY* genes from *Panax quinquefolius*, was transformed into *Arabidopsis thaliana*. In transgenic *Arabidopsis* lines, the expression level of some genes involved in triterpene biosynthesis was expressed one to five folds higher than the control lines. Meanwhile, in the transgenic lines, the expression level of some genes involved in the anti-stress process was also up-regulated [33].

Although SmWRKYs have been identified and characterized in *S. miltiorrhiza* [34], minimal information is available for illuminating the relationship between *SmWRKY* genes and those medicinal components. Methyl jasmonate (MeJA) is a key elicitor of the secondary metabolites’ biosynthetic pathway, primarily through the induction of WRKY and other TFs [35]. Thus, there may be some SmWRKYs that operate as a bridge between the MeJA signal with tanshinones and phenolic acid in *S. miltiorrhiza*. Based on this hypothesis, the hairy root of *S. miltiorrhiza* was treated by MeJA; then, we investigated the expression level of *SmWRKYs*, biosynthetic genes (*SmPAL*, *SmTAT*, *SmC4H*, *Sm4CL*, *SmHPPR*, *SmRAS*, *SmCYP98A14*, *SmDXS1*, *SmDXS2*, *SmHMGR*, *SmDXR*, *SmGGPPS*, *SmCPS*, *SmKSL*, and *SmCYP76H1*), and the accumulation of secondary metabolites. In addition, we combined the bioinformatic character of SmWRKYs, such as physicochemical properties, the phylogenetic tree, and motif analysis. Finally, those results enabled us to select high-probability SmWRKYs, which regulate tanshinones and phenolic acid biosynthesis.

## 2. Results

### 2.1. Identification of WRKY Genes in the S. miltiorrhiza Transcriptome

A total of 69 putative *SmWRKYs* were obtained from *S. miltiorrhiza*. *SmWRKYs* sequence alignment showed 61 putative *SmWRKYs* identical to Li et al. [34]. So, the 61 identical WRKYs were named by Li et al., and the others were named SmWRKY62–69, respectively. Sequences analysis of 69 identified *SmWRKYs* demonstrated the deduced amino acid numbers from 129 to 706. These SmWRKYs show a wide range of isoelectric points (pI) ranging from acidic 4.76 to very basic 9.9, suggesting extensive distribution in different microcellular environments. The molecular weights (mw) of these proteins ranged from 19.9 to 76.2 kDa. Sequence analysis of 69 WRKY protein revealed that most of WRKY proteins have conserved motif “WRKYGQK”, while four SmWRKYs (*SmWRKY14*, *SmWRKY43*, *SmWRKY63*, and *SmWRKY64*) possess WRKYGEK, and SmWRKY68 had WRKYGEK, suggesting WRKYs had diversity structure, which is and consistent with their diversity function in *S. miltiorrhiza*. The detail information of SmWRKYs were shown on the Table S1.

### 2.2. Phylogenetic Analysis of the S. miltiorrhiza WRKY Family

**Figure 1:**
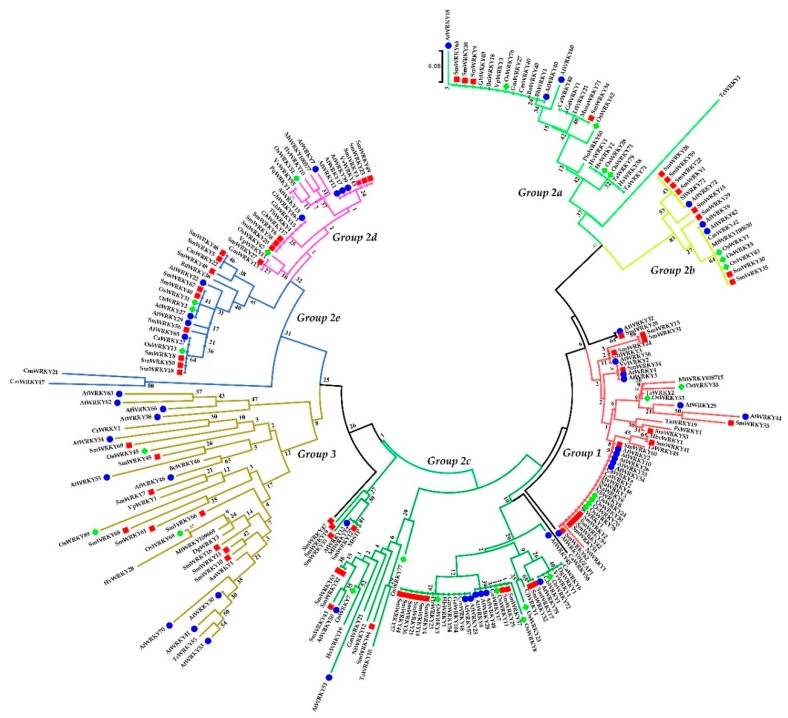
Phylogenetic tree of the WRKY from *S. miltiorrhiza* and other plants. The tree was constructed from amino sequences using MEGA 6.0 by the neighbor-joining (NJ) program with 1000 bootstrap replicates. Clades with different colors represent diverse group/subgroup.

To gain detailed knowledge of evolutionary relationship and the topological structure of the *S. miltiorrhiza* WRKY protein family, a neighbor-joining (NJ) phylogenetic tree was constructed for the WRKY domains (Figure 1), which contained 69 *S. miltiorrhiza* WRKY genes and 159 other species WRKY genes that had been studied in recent years. Based on the number of WRKY domains and the features of the specific zinc-finger motifs, all 69 *SmWRKY* genes were classified into three main groups, with five subgroups in the Group 2 [36]. 13 *SmWRKYs* genes (Thirteen: *SmWRKY2*, *SmWRKY13*, *SmWRKY24*, *SmWRKY31*, *SmWRKY39*, *SmWRKY41*, *SmWRKY42*, *SmWRKY51*, *SmWRKY53*, *SmWRKY54*, *SmWRKY55*, *SmWRKY60*, and *SmWRKY62*) with two WRKY domains were assigned to Group 1, which had a zinc-finger motif. The other 46 *SmWRKYs* genes with the zinc-finger structure of C_2_H_2_ belong to Group 2, which is comprised 66.7% of the total number of *SmWRKY* genes. The 46 Group 2 *SmWRKYs* genes are unevenly distributed amongst in five subgroups: Group 2a (four: *SmWRKY9*, *SmWRKY34*, *SmWRKY58*, and *SmWRKY65*), Group 2b (eight: *SmWRKY1*, *SmWRKY15*, *SmWRKY22*, *SmWRKY26*, *SmWRKY29*, *SmWRKY30*, *SmWRKY35*, and *SmWRKY59*), Group2c (Eighteen: *SmWRKY4*, *SmWRKY12*, *SmWRKY14*, *SmWRKY17*, *SmWRKY19*, *SmWRKY21*, *SmWRKY25*, *SmWRKY32*, *SmWRKY33*, *SmWRKY36*, *SmWRKY37*, *SmWRKY43, SmWRKY44*, *SmWRKY47*, *SmWRKY52, SmWRKY57, SmWRKY63,* and *SmWRKY64*), Group 2d (seven: *SmWRKY3*, *SmWRKY6*, *SmWRKY20*, *SmWRKY23*, *SmWRKY27*, *SmWRKY38*, and *SmWRKY49*), and Group 2e (nine: *SmWRKY5*, *SmWRKY8*, *SmWRKY18*, *SmWRKY40*, *SmWRKY46*, *SmWRKY48*, *SmWRKY50*, *SmWRKY56*, and *SmWRKY67*). In contrast, Group 2 gene has only one WRKY domain. Group 3 genes contain one WRKY domain and a C_2_HC zinc-finger motif. Nine of the 69 *SmWRKYs* genes (*SmWRKY7*, *SmWRKY10*, *SmWRKY11*, *SmWRKY16*, *SmWRKY45*, *SmWRKY61*, *SmWRKY66*, *SmWRKY68*, and *SmWRKY69*) belong to this group. Finally, *SmWRKY28*, which has two WRKY domains, does not group into Group 1, possibly because of its apparently incomplete structures. Detailed information about the category of the SmWRKYs, as well as the features of WRKY domains and zinc-finger motifs, can be found in Supplementary Tables S1 and S2, respectively.

### 2.3. Motif Composition Analysis of SmWRKY Proteins

**Figure 2:**
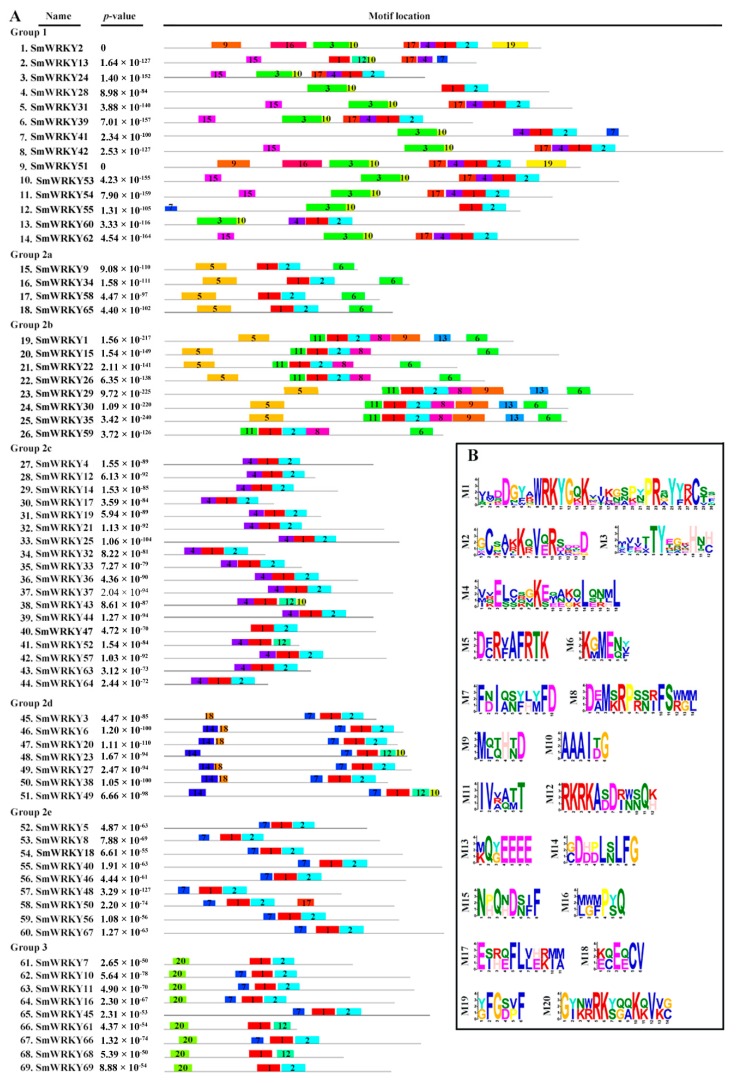
Schematic diagram of conserved motifs in WRKY proteins of *S. miltiorrhiza*. (**A**) The number symbols in colored boxes (1–20) represent different motifs. Box size indicates the length of motifs; (**B**) sequence logo of twenty conserved motifs.

To better understand the similarity and diversity of the motif compositions among different SmWRKYs, the motifs in SmWRKY protein sequences were predicted using the MEME online software. There were 20 distinct motifs that were identified (Figure 2), and the length of motifs contained 6 to 30 amino acids, and the number of motifs in each WRKY varied between 3 and 9. As shown in the Figure 2, only two motifs (motif 1, 2) were shared by every group. Most SmWRKYs in the same group had similar motif compositions. For example, motif 5 and 6 only appeared in Group 2a and 2b; motif 14 and 18 appeared exclusively in Group 2d; and motif 11 found in Group 2b. Interestingly, motif 4 only existed in Group 1 and 2c, two close subgroups in the phylogenetic tree. A more recent study, however, determined that the ancestral WRKY gene most likely encodes a Group 1 and 2c genes [37,38,39].

### 2.4. Tissue-Specific Expression of SmWRKYs

**Figure 3:**
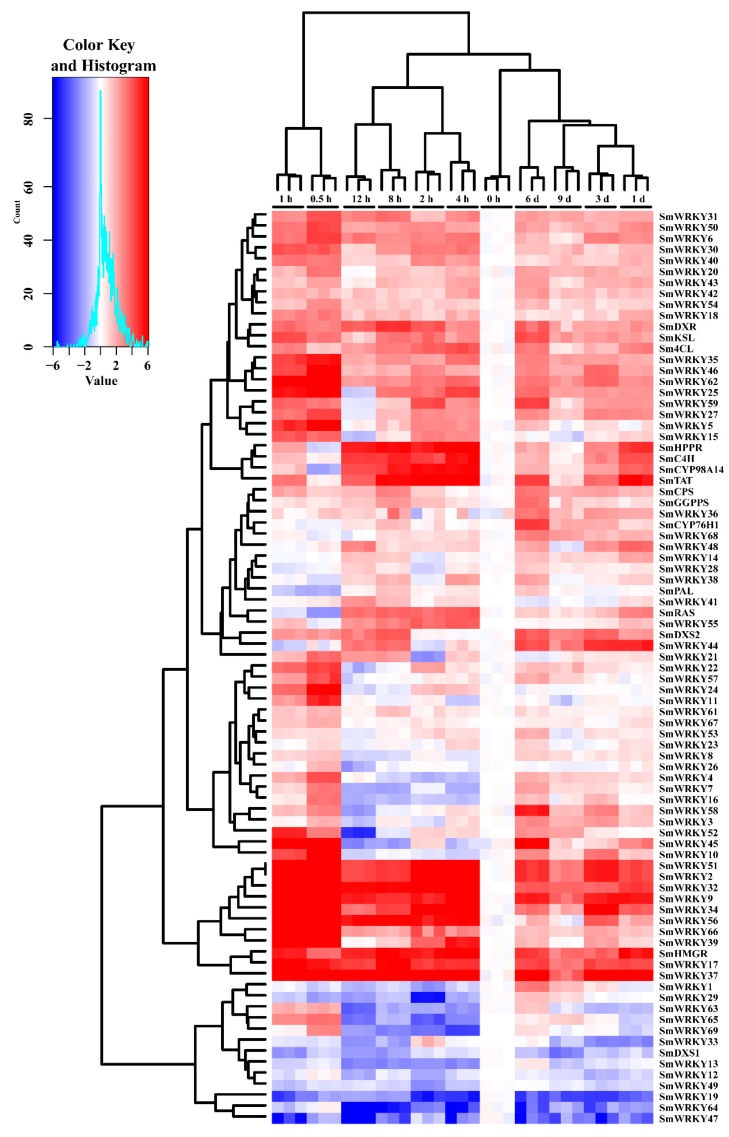
Expression of *SmWRKY* genes and biosynthetic genes in different tissues and flowering phase. Hierarchical clustering and expression profiles of the *SmWRKYs* and biosynthetic genes in different organs/tissues in *S. miltiorrhiza*. Blocks with colors indicate low/down expression (blue), high/up expression (red), or no expression/no change (white).

Accumulating evidence has revealed multiple roles of WRKY factors in response to abiotic stresses and biotic stress, secondary metabolic, and pant developmental regulation [40,41]. In order to study the potential role of SmWRKYs, we mine RT-qPCR dates that record the gene expression levels of distinct tissues including roots, stems, leaves, flowers’ varying growth stages of *S. miltiorrhiza,* and a month old seeding. Using those expression data, unsupervised agglomerative hierarchical clustering of 69 *SmWRKY* gene and 15 biosynthetic genes was constructed (Figure 3). As shown in Figure 3, it revealed five primary clusters: a flower cluster, a root cluster, a leaf and seeding cluster, and a stem cluster. Cluster of plant tissue indicated a greater difference between tissues than growth stages. However, clear differences also exist between May and October in the tissues of some genes. For example, the gene expression level in October flower of SmWRKY14 is higher than May flower. Cluster of gene expression revealed some SmWRKY genes were clustered with biosynthetic genes. Those SmWRKYs may be important for secondary metabolism accumulation by regulating biosynthetic genes, such as SmWRKY19, SmWRKY46, SmWRKY59, and so on. Interestingly, one group of SmWRKYs may have similar expression and cluster together in Figure 3, for example, SmWRKY2, SmWRKY24, SmWRKY31, SmWRKY51, and SmWRKY62 are Group 1 members (Figure 1), which are clustered together in Figure 3. In addition to SmWRKY, biosynthetic genes, such as SmHPPR, SmRAS, and SmC4H, also have similar expression pattern in different tissues. Those data suggest that similar expression pattern genes may have similar functions; therefore, we can use this character to mine some SmWRKYs that participate in secondary metabolism.

### 2.5. Differential Expression of SmWRKY Genes in Response to MeJA

**Figure 4:**
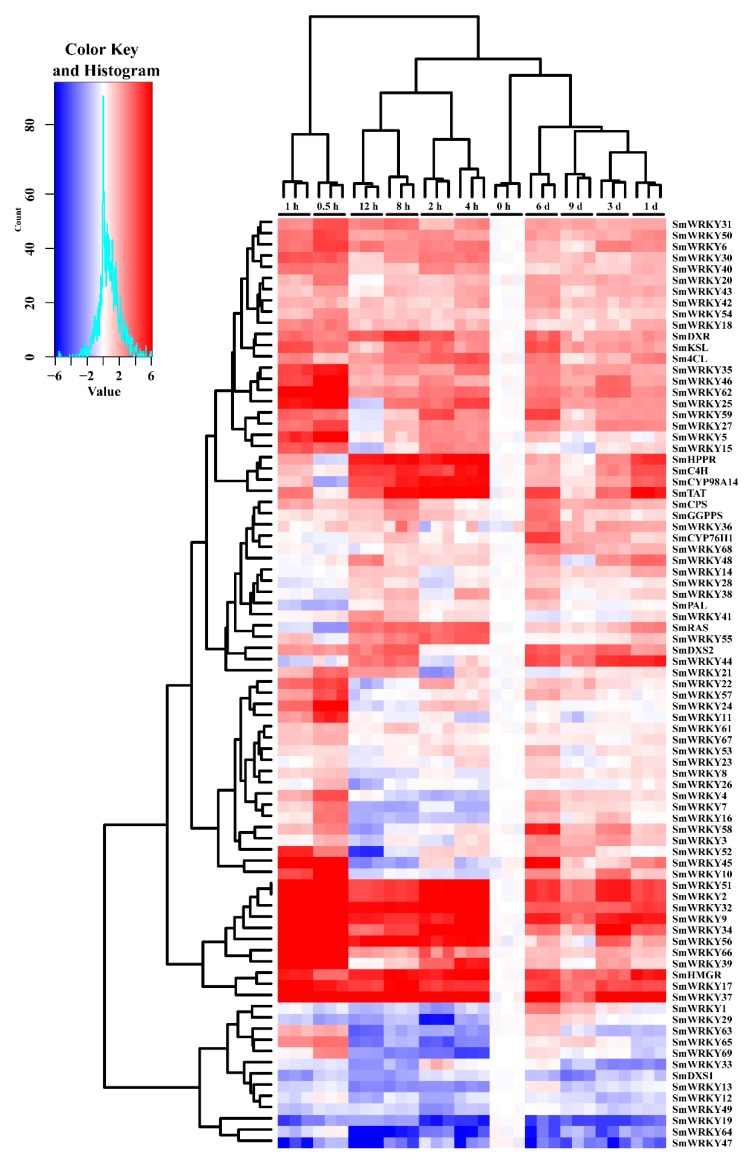
Expression of SmWRKY genes and biosynthetic genes in response the MeJA. Hairy roots of *S. miltiorrhiza* were treated by MeJA with final concentration of 100 μM for different times, and gene expression was measured via RT-qPCR analysis. The 0 h time point was used as control. Fold change in transcript abundance is illustrated as heat map on a natural log scale (treatment/control). Blocks with colors indicate low/down expression (blue), high/up expression (red), and non-expression/ no change (white).

As a key signaling molecule, MeJA plays a coordinating role in plant responses to diversity stress and in mediating secondary metabolite biosynthesis in many plant species, including *S. miltiorrhiza* [6,34,42,43]. More than 50 percent of AtWRKYs gene expression were regulated by MeJA in *Arabidopsis*. More than 1/3 of *CrWRKY* responsed to MeJA in hairy roots of *Catharanthus roseus* [40]. In order to investigate whether SmWRKY genes were responsive to MeJA, the hair roots of *S. miltiorrhza* were treated by MeJA with final concentration of 100 μM; then, the expression level of SmWRKY gene and biosynthetic genes were analyzed using the RT-qPCR method. Using these data, a hierarchical clustering (Figure 4) was constructed using unsupervised agglomerative hierarchical clustering method. As shown in the Figure 4, clustering the gene expression of treatment time revealed three primary clusters: 0.5–1 h cluster, 2–12 h cluster, and 1–9 d cluster, suggesting the gene response to MeJA was step by step. Clustering of the gene expression showed three primary clusters: About 40% of the genes of cluster one were up-regulated at different time points. The secondary cluster of genes was either up-regulated or down-regulated at different time points. Members of this cluster may indirectly respond to MeJA, and they may be regulated by another regulator. About 20% of the genes in third cluster were down regulated compared to 0 h. Meanwhile, some biosynthetic genes with similar expression pattern were clustered together, for example, phenolic acid biosynthetic genes SmHPPR, SmC4H, SmCYP98A14, SmTAT, suggesting they may have the same regulation mechanism by MeJA. In addition to those biosynthetic genes, some SmWRKY genes share similar expression patterns with biosynthetic genes, for example, SmWRKY68 was clustered with SmCYP76H1, suggesting SmWRKY68 may regulate SmCYP76H1.

### 2.6. MeJA-Induced Changes in Secondary Metabolism Biosynthesis

**Figure 5:**
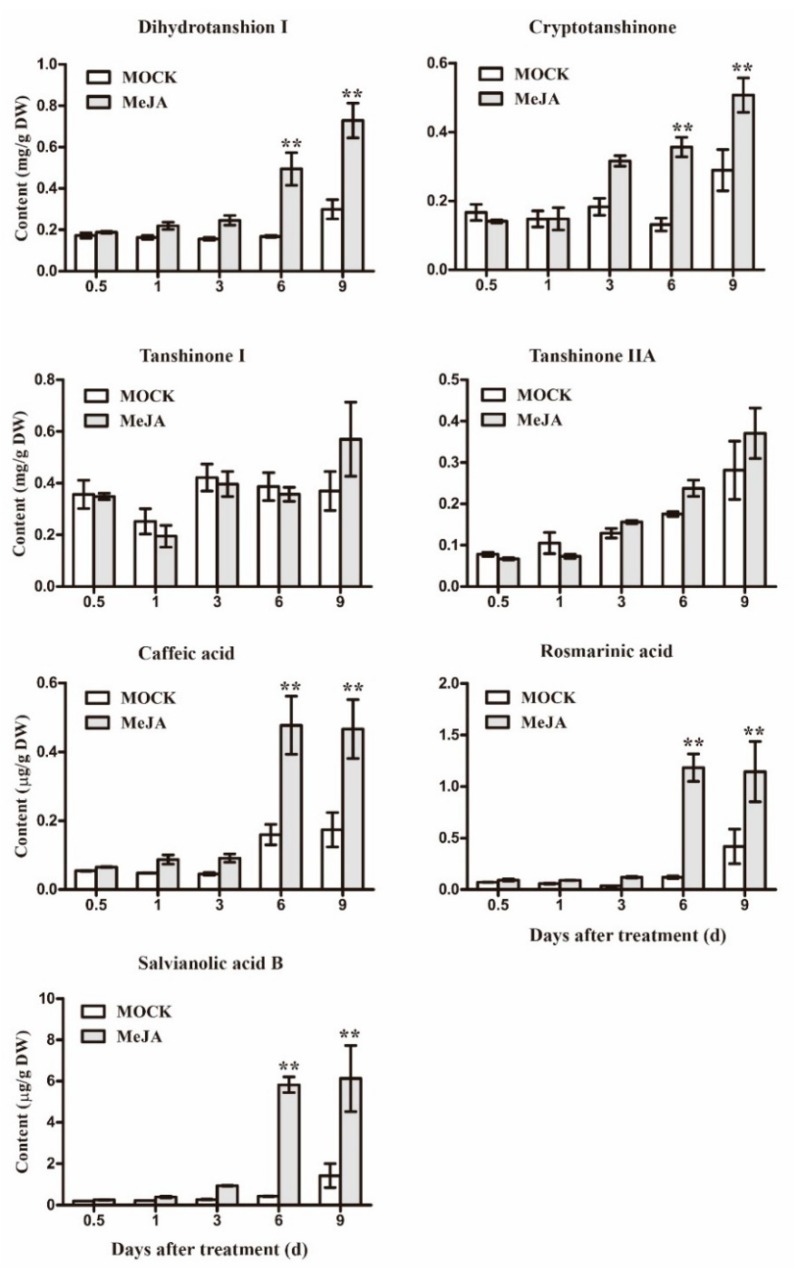
Effects of MeJA on accumulations of tanshinones and phenolic acids in *S. miltiorrhiza* hairy roots. The vertical bars show the SD values (*n* = 3). The asterisks indicate statistically significant differences at *p* < 0.05 (** *p* < 0.01) between the content in the MeJA treated cultures and that in the corresponding controls.

Hairy root of *S. miltiorrhiza,* which is regarded as a promising system with which to produce tanshinones and salvianolic acids [7,44]. As shown above, biosynthetic gene had different expression level by final concentration of 100 μM MeJA treatment. Then, we want to know the accumulation of secondary metabolism under these environments. 18 d old hair roots were treated by MeJA, and the contents of tanshinones and phenolic acids in different time points were tested by HPLC. The effects of MeJA on these metabolites were described in Figure 5. Four tanshinones (Tanshinone I, Tanshinone 2a, Dihydrotanshinone I, and Cryptotanshinone) and three phenolic acids (Caffeic acid, Rosmarinic acid, and Salvianolic acid) were investigated. The content of Dihydrotanshinone I, Cryptotanshinone, Caffeic acid, Rosmarinic acid, and Salvianolic acid were obviously elevated by MeJA treatment after 6 d and reached highest point at 9 d. However, the content of Tanshinone I and Tanshinone 2A are shown to be indistinctive by MeJA treatment, suggesting that tanshinone biosynthetic pathways were very complex.

### 2.7. Integration of Gene Expression Levels and Metabolites Accumulation Analysis

The expression level of biosynthetic gene, SmWRKY gene, and the accumulation pattern of secondary metabolite have been investigated. Then, we integrated these three parts data to predicate some SmWRKYs, which may be involved in tanshinones or phenolic acid biosynthesis. A correlation analysis using Pearson’s correlation coefficient was performed to identify possible correlation between the transcript profiles of the 69 SmWRKYs and 15 biosynthetic genes, and the 7 representative metabolites. The variable correlation coefficient cut off values of 0.6 were applied. For example, the variable correlation coefficients between SmWRKY1 expression pattern and accumulation of four tanshinones (Dihydrotanshinone I, Cryptotanshinone, Tanshinone I, and Tanshinone IIA) were 0.624, 0.648, 0.244, and 0.608, respectively. This finding indicated that SmWRKY1 as a regulator may be involved in Dihydrotanshinone I, Cryptotanshinone, and Tanshinone IIA production, but it was not correlated with Tanshinone I. Detailed correlation analyses among SmWRKY gene, biosynthetic genes, and secondary metabolites were demonstrated on Table S3. In summary, the performed study resulted in the following observation.

Members of the Group 1 (SmWRKY2, 31, 41, 42, 51, 53, 54, and 55) were correlated with not only tanshinone biosynthetic genes but also phenolic acid genes. Besides those Group 1 members, the other Group member SmWRKY62 was only correlated with tanshinone biosynthetic gene. Among the Group 2 family, Group 2a member SmWRKY9 was correlated with both tanshinone and phenolic acid biosynthetic genes. In addition, the other Group 2a member 58 was only correlated with *SmDXS2*, *SmGGPPS*, *SmCPS*, *SmKSL*, and *SmCYP76H1*, which were tanshinone biosynthetic pathway genes. SmWRKY30 and SmWRKY59, Group 2b member, were significantly correlated with tanshinone and phenolic gene biosynthetic genes, while other Group 2b members SmWRKY35 and SmWRKY30 were correlated with tanshinone biosynthetic genes and phenolic acid genes, respectively. Group 2c members (SmWRKY14, 17, 32, 36, 37, 43, and 47) showed simultaneous correlation with tanshinone and phenolic acid biosynthetic genes. In addition to this, SmWRKY25 and SmWRKY44 were highly correlated with tanshinone biosynthetic genes, while SmWRKY21, 33, and 63 were correlated with phenolic acid genes, especially downstream of phenolic acid biosynthetic pathway genes. Group 2d members, SmWRKY6, 23, and 38, were highly correlated with both tanshinone and phenolic acid biosynthetic genes. Group 2e members, SmWRKY5 and SmWRKY40, were correlated with tanshinone biosynthetic genes, while SmWRKY8 and SmWRKY18 were correlated with phenolic acid biosynthetic genes. Interestingly, in the correlation analysis level, SmWRKY5 and SmWRKY play opposite roles in tanshinone biosynthesis. Among the Group 3 family, SmWRKY45 and SmWRKY61 were simultaneously correlated with tanshinone and phenolic acid biosynthetic genes. The other group members, SmWRKY16 and SmWRKY66, were highly correlated with phenolic acid and tanshinone biosynthetic genes, respectively. SmWRKY28, which was not classified into any group, was simultaneously correlated with tanshinones and phenolic acid biosynthetic genes.

Additionally, SmWRKYs were correlated with secondary metabolism biosynthetic genes; SmWRKY1, 7 29, 52, and 56 displayed high correlation with phenolic acid metabolites. SmWRKY1, 7, 19, 29, 52, 56, and 58 were highly correlated with not only tanshinone biosynthetic genes but also tanshinone metabolites. Meanwhile, SmWRKY45, 58, and 68 displayed high correlation with phenolic acid biosynthetic genes and their metabolites. Based on these analysis, nine SmWRKYs, namely, SmWRKY1, 7, 19, 29, 45, 52, 56, 58, and 68, are most likely involved in the biosynthesis of secondary metabolites in *S. miltiorrhiza.*

## 3. Discussion

*S. miltiorrhiza* is one of the most important medicinal plants in China. The tanshinones and phenolic acids were main active pigments that were produced by different biosynthetic pathways. Most biosynthetic enzyme genes have been cloned in recent years [5,6]. However, the research of these component regulatory mechanisms at the molecular level, especially at the transcription factor regulation level, was limited. WRKY is a key regulator that plays diverse roles in plant growth and development, in response to various stresses. In addition, WRKY also plays important role in secondary metabolism biosynthesis. Although Li et al. have been genome-wide characterized, molecularly cloned, and expression analyzed by WRKY in *S. miltiorrhiza* [34], rare information has been provided by this key transcription factor in tanshinones and phenolic acid biosynthesis. 

Therefore, we need to carry out an in-depth analysis of the *S. miltiorrhiza* WRKY family. In this study, 69 SmWRKYs have been identified by integrating multiple transcriptome databases. The WRKY TFs was named by its N-terminal conserved amino acid sequence WRKYGQK. However, multiple sequence alignments of these SmWRKYs revealed 5 SmWRKY proteins (SmWRKY14, SmWRKY43, SmWRKY63, SmWRKY64, and SmWRKY68) had sequence variations in their WRKY domain. For example, SmWRKY43, the WRKY-conserved domain amino acid sequence, has been replaced by WRKYGKK. The subtle variation in WRKY-conserved domain may recognize non-W-box element. For instance, the tobacco NtWRKY12, which contains a WRKYGKK motif, binds to WK-box (TTTCCAC), which is substantially different from the W-box shown to be recognized by WRKYGQK motif [45]. Moreover, soybeans, GmWRKY6 and GmWRKY21, which also contain a WRKYGKK motif, do not bind normally to the W-box [46]. It therefore seems that variations in the WRKYGQK motif might alter their DNA binding affinity, and so it would be interesting to investigate the functions and binding specificities of SmWRKY14, SmWRKY43, SmWRKY63, SmWRKY64, and SmWRKY68. Moreover, as far as is known, nothing is known about the effect of zinc-finger motif changes [47].

Structure diversity determines its function diversity. Hence, WRKYs have different roles in plants. However, the main aim of the present research was to identify potential SmWRKY transcription factors involved in tanshinones and phenolic acids biosynthesis. MeJA plays an important role in enhancing the production of secondary metabolite; for example, tabersonine, ajmalicine, and catharanthine showed dramatic accumulation after MeJA induction in *Catharanthus roseus* seedlings [48]; lariciresinol, secoisolariciresinol, and pinoresinol as key bioactive ingredients in *Isatis indigotica*, which were also enhanced by MeJA treatment [49]. In *S. miltiorrhiza*, dihydrotanshinone I, cryptotanshinone, caffeic acid, rosmarinic acid, and salvianolic acid B were increased by MeJA (Figure 4). How do MeJA steer secondary metabolism production? Many transcription factors, usually induced by MeJA, with a role in the MeJA-modulated regulation of secondary metabolism were discovered, such as WRKY, bHLH, and MYC2 [50]. Therefore, there may be a “bridge” in connection with transcription factor, biosynthetic gene, and secondary metabolite. Based on this hypothesis, gene expression and metabolite analysis were integrated to elect key gene involved in active pigment produce. In this method, Chen and his coworkers discovered four putative AP2/ERFs (li08, 007, 049, and 050) that showed high probability of being involved in the regulation of lignin production [49]. As a result, we found that SmWRKY1, 7, 19, 29, 45, 52, 56, 58, and 68 were most likely involved in the secondary metabolism production of *S. miltiorrhiza*. 

In addition to using a statistical method to identify potential biosynthetic genes, phylogenetic tree also can be assisted in target gene mining. SmWRKY1 and SmWRKY29, Group 2b member, were phylogenetically related to MtWRKY100630 from *Medicago truncatula* (Figure 1) and might have a functionality in increasing the level of caffeic acid [51]. Our research was consistent with these predictions, because SmWRKY1 and SmWRKY 29 were highly correlated with caffeic acid element, and the correlation coefficients were 0.756 and 0.705, respectively (Table S3). Group 3 members SmWRKY7 and SmWRKY68 also might be involved in secondary metabolism biosynthesis under correlation analysis. As shown in the Figure 1, SmWRKY7 and SmWRKY68 were phylogenetically related to AaWRKY1, OsWRKY45, and OsWRKY80. AaWRKY1 was a key regulator of artemisinin biosynthesis. OsWRK45 plays a central role in salicylic acid signaling pathway against diseases, and overexpression of *OsWRKY45* can enhance diterpenoid phytoalexins production [52]. OsWRKY89, induced by MeJA and UV-B radiation, plays an important role in lignin biosynthesis. Based on these findings, SmWRKY7 and SmWRKY68 may not only be involved in metabolites biosynthesis but also steered *S. miltiorrhiza* against biotic and abiotic stresses. Further, 20 different types of motif were found among 69 SmWRKY, suggesting that in order to perform diversified functions, these WRKYs have acquired additional domains, which leads to variations in their structural and physiological features [53].

## 4. Materials and Methods

### 4.1. Identification of SmWRKY Genes Family Members

The nucleotide sequences and amino acids of 72 AtWRKY genes were downloaded from the Arabidopsis Information Resource (TAIR; Available online: http://www.Arabidopsis.org/). The transcriptome of *S. miltiorrhiza* were assembled using public data from SRA database (Available online: http://trace.ncbi.nlm.nih.gov/Traces/sra/), including SRX360510, SRX317054, SRX317052, SRX224100, SRX021907, SRX021907, SRX017265, and Danshen Transcriptional Resource Database (DsTRD, Zhejiang Sci-Tech University, Hangzhou, China) [54]. We used *Arabidopsis thaliana* WRKY sequence as query for BLAST searches against those *S. miltiorrhiza* databases. The sequences were selected as candidate sequences for further study if their *E* value was ≤e^−10^. Candidate sequences were confirmed for presence of WRKY domains by use of the HMMER 3.0 (Available online: http://hmmer.org/). The WRKY-like sequences confirmed by Hmmersearch in the *S. miltiorrhiza* database were in turn used reiteratively to search SmWRKY until no new sequences were found. The WRKY sequences in different transcriptome databases were blasted using Cluster 3.0 software (Laboratory of DNA Information Analysis Human Genome Center, Institute of Medical Science, University of Tokyo, Tokyo, Japan), and those having same WRKY core domain with 60 amino acids were considered as one WRKY gene. The theoretical isoelectric point (pI) and molecular weight (mw) of full-length CDS of SmWRKY were predicted using the Compute pI/Mw tool on the ExPASy server (Available online: http://web.expasy.org/compute_pi/) [55].

### 4.2. Multiple Sequence Alignment, Phylogenetic Analysis

Multiple sequence alignment of the WRKY domain from *S. miltiorrhiza* SmWRKYs, *Arabidopsis* AtWRKYs, and other pants WRKYs that were reported in its functions in recent years were performed using CLUSTALW, and phylogenetic trees were constructed using the neighbor-joining method (Bootstrap test was replicated 1000 times) using MEGA 6.0 software. All the SmWRKYs were analyzed by MEME online software (Available online: http://meme-suite.org/tools/meme) for conversed motif prediction with the following criteria: 20 motifs, with an optimum motif width between 8 and 50 residues, with any number of repetitions. The sequences for phylogenetic trees and conversed motif analysis are shown in Tables S1 and S2.

### 4.3. Materials and Hairy Root Culture with MeJA Treatment

Flowers, leaves, stems, and roots of *S. miltiorrhiza* plants, grown in field (Zhejiang Sci-Tech University Medicinal Herb Garden, Hangzhou, China) for 2 years, were collected in May and October 2015, separately, and seedlings grown for one month in greenhouse were also sampled. Species verification was identified by Dongfeng Yang of Zhejiang Sci-Tech University.

Hairy-root culture system of *S. miltiorrhiza* was referred to Liang et al. [56] and Yang et al. [56]. 250 mL triangular flask was carried out on an orbital shaker running at 110–120 rpm in darkness. Each flask with 50 mL hormone-free 6,7-V liquid medium was inoculated with 0.3 g fresh hairy roots cultured for 3-weeks at 25 °C, and 100 mM MeJA [57] was added to the root culture on day 18 post-inoculation. Untreated hairy roots were designated as the control. Hairy roots at 0.5, 1, 2, 4, 8, 12, 1, 3, 6, and 9 d were sampled. All the plant samples have three independent bio-repeats, and each sample was divided into two parts for RNA extraction and metabolite content analysis.

### 4.4. Transcriptional Analysis by Real-Time Quantitative PCR (RT-qPCR)

The samples stored in liquid nitrogen were used for RNA extraction. Total RNA was isolated from organs and hairy roots using the RNAprep pure Plant Kit (Tiangen, Beijing, China) and then reversely transcribed according to the manufacturer’s instruction of PrimeScript™ RT reagent Kit (Takara, Shiga, Japan). RNA integrity was analyzed on a 1.0% agarose gel. RNA quantity was determined using a NanoDrop 2000 Spectrophotometer (Thermo Scientific, Woburn, MA, USA). The obtained cDNA was used as template for the RT-qPCR analysis using QuantStudio™ Flex6 System (ABI, Alexandria, VA, USA) with SYBR green reagents (Takara, Dalian, China). The primers are in Supplementary Table S4. *SmUBQ10* [6] was used as an internal control. RT-qPCR was performed according to the following condition: 30 s pre-denaturation at 95 °C, and 40 cycles with 5 s at 95 °C and 30 s at 59 °C. Experiments were performed in triplicate for each bio-repeat, and the results were represented by their means ± SD. Quantification of the gene expression was done with 2^−∆∆*C*t^ method [58].

### 4.5. Metabolite Extraction and HPLC Analysis

Metabolite extraction and analysis followed the methods [59,60] with minor modifications. The dried plant organs and hairy roots were ground to powder with mortar and pestle. 50 mg sample powder was extracted with 5 mL 70% methanol under sonication for 45 min, and then centrifuged at 8000 rpm (6010× *g*) for 10 min. The supernatant was used for HPLC analysis. Waters HPLC system (Milford, MA, USA) contained a 1525 binary pump, an automatic sample injector, and a Waters 2998 photodiode array detector (PDA). HPLC separation was performed with a SunFire C18 column (4.6 mm × 250 mm, 5 μm particle size) at 30 °C. Empower 3 software (Milford, MA, USA) was used for data acquisition and analysis. The sample injection volume was 20 μL; the PDA detection wave length for the lipid-soluble tanshiniones was 270 nm, and for the water-soluble phenolic acids it was was 280 nm. Separation was achieved by elution using a linear gradient with solvent-A (acetonitrile) and solvent-B (0.026% phosphoric acid solution). The gradient was as follows: 0–10 min, 5–20% A (*v*/*v*); 10–15 min, 20–25% A (*v*/*v*); 15–20 min, 25% A (*v*/*v*); 20–25 min, 25–20% A (*v*/*v*); 25–28 min, 20–30% A (*v*/*v*); 28–40 min, 30% A (*v*/*v*); 40–45 min, 30–45% A (*v*/*v*); 45–58 min, 45–58% A (*v*/*v*); 58–67 min, 58–50% A (*v*/*v*); 67–70 min, 50–60% A (*v*/*v*); 70–80 min, 60–65% A (*v*/*v*), and 80–85 min, 65–100% A (*v*/*v*). Standards of metabolite compounds were purchased from the National Institute for the Control of Pharmaceutical and Biological Products (Beijing, China). A series of the standard solutions (0.02, 0.05, 0.1, 0.2, 0.5, and 1 mg/mL) of tanshinones and phenolic acids were used to make standard curves. The regression equations and correlation coefficients were generated by three repeats: *y* = 3,387,376*x* − 39,835, *R*^2^ = 0.9997, for dihydrotanshinone I; *y* = 3,229,227*x* + 29,146, *R*^2^ = 0.9988, for cryptotanshinone; *y* = 1,806,477*x* + 20,298, *R*^2^ = 0.9959, for tanshinone I; *y* = 5,190,705*x* + 9846, *R*^2^ = 0.9997, for tanshinone IIA; *y* = 4,237,498,693*x* + 2,758,119, *R*^2^ = 0.9990, for caffeic acid; *y* = 1,714,450,161*x* + 2,808,215, *R*^2^ = 0.9998, for rosmarinic acid; *y* = 1,077,835,588*x* − 21,701, *R*^2^ = 0.9990, for salvianolic acid B. *x* stands for the content of metabolite (mg/mL) and *y* for the peak area.

### 4.6. Data Analysis

For statistical analysis, ANOVA (analysis of variance) was calculated using SPSS (Version 19.0, IBM, Armonk, NY, USA). The differences between treatments were considered to be statistically significant when *p* values <0.05.

## 5. Conclusions

The WRKY family of *S. miltiorrhiza* was identified as possessing a total of 69 members. Structural and phylogenetic tree analysis revealed 69 SmWRKY could be divided into 3 groups (1, 2, and 3), and Group 2 could be divided into 5 subgroups (2a, 2b, 2c, 2d, and 2e). A total of 20 conserved motifs were identified, of which group-specific motifs might attribute to functional divergence of WRKYs. Many SmWRKYs were phylogenetically related to other plant WRKYs that may share the same function, suggesting those SmWRKYs may have a similar function. Gene expression profiles suggest that the majority of 69 SmWRKY genes are tissue-specific and MeJA-responsive. Meanwhile, secondary metabolite accumulation was also induced by MeJA. Based on these analysis, integration of gene expression level, metabolite accumulation, and phylogenetic tree analysis, SmWRKY1, 7, 19, 29, 45, 52, 56, 58, and 68, were most likely involved in secondary metabolism production. Taken together, our study has generated an important resource that could be used for further studies of SmWRKY genes in *S. miltiorrhiza* and especially could be used as a cue for further investigating SmWRKY functions in secondary metabolite accumulation.

## Supplementary Materials

Supplementary materials can be found at http://www.mdpi.com/1422-0067/19/6/1593/s1.

## Abbreviations

C4H	cinnamic acid 4-hydroxylase
CPS	copalyl diphosphate synthase
CYP98A14	cytochrome P450-dependent monooxygenase
CYP76AH1	cytochrome P450-dependent monooxygenase
DXR	1-deoxy-d-xylulose 5-phosphate reductoisomerase
DXS	1-deoxy-d-xylulose 5-phosphate synthase
GGPP	geranylgeranyl diphosphate
GGPPS	geranylgeranyl diphosphate synthase
HMGR	3-hydroxy-3-methylglutaryl CoA reductase
HPLC	high performance liquid chromatography
HPPR	4-hydroxyphenylpyruvate reductase
KSL	kaurene synthase-like
MeJA	methyl jasmonate
PAL	phenylalanine ammonia-lyase
PDA	photodiode array detector
RT-qPCR	quantitative real-time PCR
*S. miltiorrhiza*	*Salvia miltiorrhiza*
TAT	tyrosine aminotransferase
4CL	hydroxycinnamate coenzyme A ligase
TFs	transcription factors
MVA	mevalonate
MEP	methylerythritol phosphate
